# Survey on the current gynaecological approach of ovarian cancer patients: The utility of HIPEC

**DOI:** 10.1515/pp-2019-0029

**Published:** 2020-02-26

**Authors:** Christos Iavazzo, Alexandros Fotiou, M. Tsiatas, Athina Christopoulou, John Spiliotis, Paul Sugarbaker

**Affiliations:** Gynaecological Oncology Department, Metaxa Cancer Hospital, Piraeus, Greece; Medical Oncology Department, Athens Medical Centre, Athens, Greece; Medical Oncology Department, Saint Andrews Hospital, Patra, Greece; Surgical Oncology and HIPEC Department, European Interbalkan Medical Centre, Thessaloniki, Greece; Center for Gastrointestinal Malignancies, Washington, DC, USA

**Keywords:** hyperthermic intraperitoneal chemotherapy (HIPEC), ovarian cancer, survey

## Abstract

**Background:**

The aim of this survey was to acquire an overview of the current management of ovarian cancer with an emphasis on the utility of hyperthermic intraperitoneal chemotherapy (HIPEC).

**Methods:** An email was sent to Oncologists prior to PSOGI International Symposium on Advanced Ovarian Cancer, Athens 11–13 April 2019. Doctors submitted responses on the relevant website. The self-report survey contained 17 questions.

**Results:**

In total, 467 Medical Oncologists, Surgical Oncologists or Gynaecologic Oncologists were participated and answered to this survey. The resectability of disease was evaluated by laparoscopy from 48.5% of the participants, while 51.5% answered that they stage their patients pre-surgically with the use of CT or MRI. The preferred first intervention in advanced ovarian cancer patients is the neoadjuvant chemotherapy followed by interval cytoreductive surgery (72%). Regarding the use of HIPEC, almost half of the participants answered that there is role of HIPEC use in ovarian cancer patients undergoing interval debulking surgery, while almost 70% answered positively about the utility of HIPEC use in ovarian cancer recurrence. As for the role of lymphadenectomy in advanced ovarian cancer patients, half of the responders answered negatively. Finally, only 25% of the participants responded that they always check the *BRCA* status of their ovarian cancer patients, despite the possible differentiation of treatment based on the molecular profiling (80%).

**Conclusions:**

The results of this survey indicate the utility of HIPEC in treatment of ovarian cancer patients and the differences in the overall management of ovarian cancer patients in the current clinical practice.

## Introduction

Ovarian cancer (OC) accounts for an estimated 295.414 new cases worldwide, which is about 3.4% of overall new cases of cancer [1]. Annually OC is responsible for 184.799 deaths which accounts 4.4% of all deaths from cancer [1]. In the United States of America, OC accounts more deaths than all the other gynaecological malignancies except breast cancer combined [1]. Approximately 20% of new patients are diagnosed with localized tumour (FIGO stage I) with a 5-year survival rate of 92%. Generally, the overall 5-year survival rate ranges between 30–40% across the globe.

Optimal cytoreductive surgery (primary or interval) and adjuvant or neoadjuvant taxane-plus platinum-based combination chemotherapy is the gold standard treatment for patients with advanced OC [2, 3]. Complete cytoreduction is one of the most significant predictors of survival. For this reason, the 10 years aggressive ultraradical procedures are a common approach of such a lethal disease. Also, several trials are conducted regarding the new treatment options for recurrent OC [4, 5].

Although the available evidence regarding the role of hyperthermic intraperitoneal chemotherapy (HIPEC) in such patients is inconclusive at the moment, several teams are implementing it as a useful and promising tool in the armamentarium against OC. Several studies have been published with very promising results regarding the safety and utility of HIPEC in disease-free and overall survival [6, 7, 8, 9]. Regarding the morbidity and mortality of HIPEC use in OC patients several studies, both trials and meta-analyses especially the last years, reported that the association of primary or secondary cytoreductive surgery plus HIPEC is safe with an improved PDS and OS compared with cytoreduction without HIPEC [6, 8, 9, 10].

This survey study about the advanced OC and its treatment sought to understand Oncologists (Surgical, Gynaecologic or Medical) knowledge of, experience with and attitudes toward the use and the utility of HIPEC in advanced OC patients. In this article, we represent the Gynaecologic Oncologists’ perspective about the utility of HIPEC in these patients’ treatment.

## Materials and methods

We developed a 17-question, self-report survey request (Supplementary File), that was emailed to Oncologists prior to PSOGI International Symposium on Advanced Ovarian Cancer, Athens, 11–13 April 2019. Doctors submitted responses on the relevant website.

Participant’s specialties that were recruited, are medical, surgical or gynaecological oncologists.

Information obtained included specialty, sex, ethnicity, and practice patterns, as well as surgical expertise, techniques, and rationale with respect to HIPEC use in patients with advanced epithelial OC.

All subjects were asked about challenges and barriers associated with treatment of advanced OC patients. Respondents were encouraged to indicate what procedure they use in surgical treatment of these patients or what procedure they think is more effective.

The questionnaires consisted of multiple choices questions for each category. There were no open ended/free write questions included.

### Data analysis

Descriptive statistics detailing the counts and percentages was the primary statistical method used. Subgroup analyses depending the specialty of responders were conducted. Statistical analysis was performed using SPSS software program, version 24.0.

### Ethics

The study was approved by the local Ethical Committee at Athens Medical Centre. All participants provided informed consent to anonymously analyse their answers. No individualized personal data were collected.

## Results

**Figure 1:**
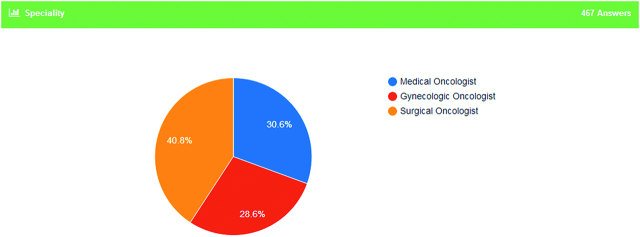
Specialty of participants.

Survey responses were completed by 467 Medical Oncologists, Surgical Oncologists or Gynaecologic Oncologists and reported as a proportion for each query. 30.6% were Medical Oncologists, 40.8% were Surgical Oncologists and 28.6% were Gynaecologic Oncologists (Figure 1). All the answers are presented in Table 1.

**Table 1:** Questions and results.

QUESTION				
1. First intervention of advanced ovarian cancer	25% optimal primary debulking	72% neoadjuvant chemotherapy followed by interval surgery	3% neoadjuvant chemotherapy followed by interval debulking and HIPEC	
2. Approach to evaluate the resectability of advanced ovarian cancer disease	48.5% upfront laparoscopy	37.1% CT-MRI findings	14.4% CT-MRI findings with experts Radiologists and MTD discussion	
3. Neoadjuvant chemotherapy based on positive cytology from ascetic fluid	47% yes	41.7% no	11.4% do not know	
4. Perform of primary debulking surgery in patients with mesenteric disease	7.6% always	76.5% sometimes	15.9% never	
5. Perform of primary debulking surgery in patients with upper abdominal disease	14.4% always	75.8% sometimes	9.1% never	0.7% do not know
6. Benefit on overall survival of extended surgical procedures	45.5% yes	54.5% no		
7. Role of pelvic/paraaortic lymph node dissection in patients with ovarian cancer	23.5% always	72% sometimes	4.5% never	
8. Role of pelvic/paraaortic lymph node dissection in fertility sparing surgery for early stage ovarian cancer	9.1% always	38.6% sometimes	52.3% never	
9. Role of HIPEC in ovarian cancer patients undergoing interval debulking surgery	3.8% always	44.7% sometimes	42.4% never	9.1% do not know
10. Value of surgery in recurrent ovarian cancer	44.7% yes	47.7% no	7.6% do not know	
11. Role of HIPEC in ovarian cancer recurrence	20.5% always	47.7% sometimes	25% never	6.8% do not know
12. Role of PIPAC in ovarian cancer patients	1.6% always	21.2% sometimes	34.8% never	42.4% do not know
13. Check of *BRCA* status in all ovarian cancer patients	25% always	66.7% sometimes	7.6% never	0.7% do not know
14. Differentiation of treatment based on molecular profiling of patients	5.3% always	73.5% sometimes	20.5% never	0.7% do not know
15. Main reason of residual disease after primary debulking surgery	59.8% mesenteric disease	20.5% disease at porta hepatis	12.1% small bowel carcinomatosis	7.6% diaphragmatic involvement
16. Prophylactic salpingectomy at benign gynecological surgery and caesarean section	18.2% yes	81.8% no		
17. Evaluation by ESGO certified Gynecological Oncologist of any suspected ovarian cancer patient	29.5% yes	69.7% no	0.8% do not know	

The largest amount of participants were European. Sixty percent of the doctors were from this continent (n=280). We subanalyzed the Gynaecological Oncologists’ answers. Seventy 5% of the responders were men, with a 25% of responders to be women.

**Figure 2:**
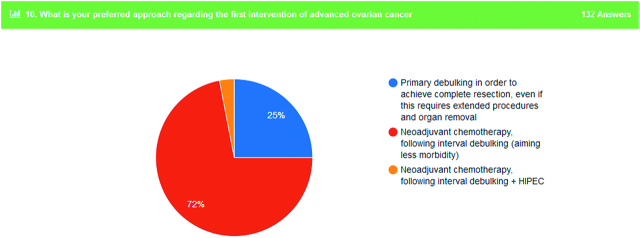
First intervention in advanced ovarian cancer patients.

Regarding the first intervention in patients with advanced OC 72% of participants answered that is preferable for the patient to undergo neoadjuvant chemotherapy and then interval debulking surgery (Figure 2). Aim of this tactic is to minimize the morbidity of an extended primary procedure. On the contrary, 25% of the responders prefer to approach these patients with a primary surgery in order to achieve complete resection.

**Figure 3:**
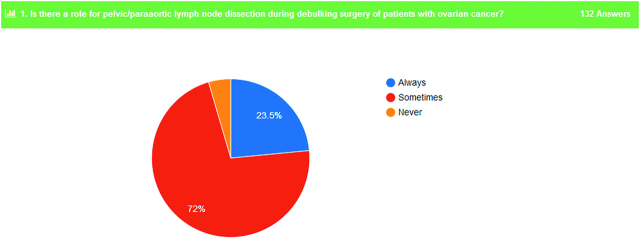
Ovarian cancer and lymphadenectomy.

**Figure 4:**
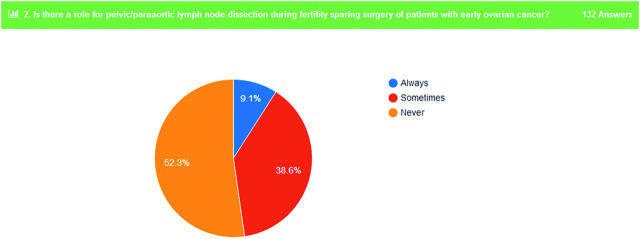
Fertility sparing surgery and lymphadenectomy.

Concerning the possible role of pelvic/paraaortic lymph node dissection during debulking surgery for OC, 95.5% of the participants responded that they do always or in some cases this procedure (Figure 3). In addition to that, 90.9% of the responders identified a possible role of pelvic/paraaortic lymphadenectomy even if the patient undergo fertility sparing surgery for early OC (Figure 4).

**Figure 5:**
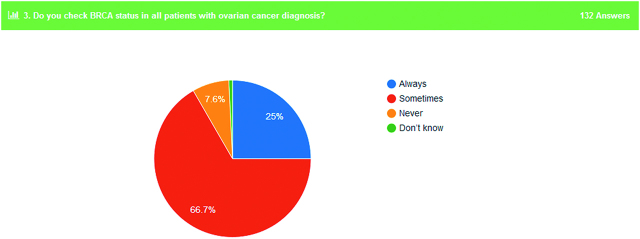
*BRCA* status investigation in ovarian cancer patients.

Surprisingly, only 25% of the participants answered that they always check the *BRCA* status of their patients and 66.7% of them responded that they sometimes check the *BRCA* status (Figure 5). However, 94% of doctors said that the treatment of ovarian cancer patients can differentiate based on molecular profile of the patient.

As for the extent of the disease and the possibility for a surgical treatment, a 16% of the participants respond that they never perform primary debulking surgery in OC patients with mesenteric disease. Even more if the patient’s disease expands to the upper abdomen; only 9% of the doctors will perform primary debulking surgery to these patients. In order to evaluate the resectability of disease in advanced stage 48.5% answered that they submit their patients in upfront laparoscopy, while 51.5% of responders prefer to stage their patients pre-surgically with the use of CT or MRI. 48% of the participants consider acceptable to administer neoadjuvant chemotherapy based on positive cytology. Only 45.5% recognize a survival benefit in patients undergoing extended surgical procedures (total peritonectomy, cholecystectomy, etc.), comparing to an elective surgical approach based on selected removal of disease. Concerning the main reason and areas of residual disease in patients undergoing primary cytoreductive surgery, mesenteric disease (59.8%) and disease at porta hepatis (20.5%) are incriminated.

**Figure 6:**
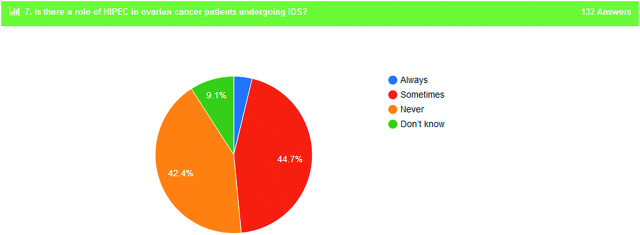
Role of HIPEC in IDS surgery.

Our main target of this survey was to evaluate the opinion of Gynaecologic Oncologist as for the utility of HIPEC in treatment of OC patients. Regarding this question, 48.5% of the responders identified a role of HIPEC in patients undergoing interval debulking surgery (Figure 6), while 68.2% of them answered positive concerning the role of HIPEC in recurrence of disease.

In addition to that, 44.7% answered that patients with recurrence can undergo to a new cytoreductive surgery. Pressurized intraperitoneal aerosol chemotherapy (PIPAC) is a highly experimental approach with only 22.8% of participants identified role in treatment of OC patients, while 42.4% are now aware at all of any evidence regarding the possible role of PIPAC.

Last but not least, only 29.5% of participants answered that a patient with a suspected OC should be evaluated by an ESGO certified Gynaecological Oncologist, while only 18.2% respond that they perform incidental prophylactic salpingectomy at benign gynaecological surgery and caesarean section.

## Discussion

We conducted a survey in order to understand Gynaecological Oncologists’ knowledge and experience with advanced OC patients. A question, that Gynaecological Oncologists who participated to this study had to answer, were what is their preferred approach regarding the first intervention to an advanced OC patient. The dominant opinion is that neoadjuvant chemotherapy, followed by interval debulking surgery is the preferred approach. This result fits with the published results of a survey from the members of ESGO about the role of neoadjuvant chemotherapy in the management of stage III–IV OC patients [11]. This subject has been studied by several published articles, showed that either neoadjuvant chemotherapy and interval surgery or primary cytoreductive surgery is an acceptable strategy, with the complete resection of all the macroscopic disease to be the main target [3]. Moreover, a recent article that analyse the pooled individual patient data from advanced OC patients that were included in two randomized trials (EORTC 55,971 and CHORUS) with 1220 patients finally included, mentioned that both strategies had similar results in overall survival in advanced OC. In addition to that, this article reported that patients with stage IV OC had a better survival with neoadjuvant chemotherapy [2]. A new international, open, randomized, controlled multi-centre trial investigating the optimal time of surgical therapy in patients with advanced OC will clarify if these patients should undergo primary cytoreductive surgery and then adjuvant chemotherapy or neoadjuvant chemotherapy and then interval surgery. Results are expected in 2024 [12].

Another controversial field in treatment of advanced OC patients is the role of lymph node dissection, pelvic and/or paraaortic in primary debulking surgery or after adjuvant chemotherapy. Lymph node metastasis has a significant contribution to the prognosis of epithelial OC but the role of lymph node dissection in treatment is controversial. Prior published articles have demonstrated the potential importance of lymph node dissection for the detection of occult metastases with the main condition of complete macroscopic cytoreduction [13, 14]. A recent retrospective study mentioned that dissection of more than 10 lymph nodes is a significant prognostic parameter. This article, also, referred a longer survival in patients underwent paraaortic and pelvic lymph node compared to patients underwent only pelvic lymphadenectomy and an improved progression free survival in patients underwent lymphadenectomy [15]. Moreover, a study observed significant beneficial effects of systematic lymph node dissection during primary debulking surgery on both progression free and overall survival in patients with negative lymphadenopathy on a preoperatively CT scan [16]. All these comes to contrary with the results of LION trial. LION study randomised 647 patients with advanced OC undergoing complete resection and had no bulky lymph nodes both before and during surgery to either undergo or not systematic pelvic and/or paraaortic lymphadenectomy. The median overall survival was 69.2 months in the no-lymphadenectomy group and 65.5 months in the lymphadenectomy group while median progression-free survival was 25.5 months in both groups. This trial suggests that lymph node dissection did not increase the overall or the progression-free survival. In addition to that, lymph node dissection patients presented increased rate of postoperative complications [17].

One other question in our survey was the role of lymphadenectomy in patients underwent fertility sparing surgery for early stage OC. Approximately 48% of participants identified a positive role of this procedure. Although the majority of the cases are diagnosed on advanced stage, a minor proportion is identified in young nullipara patients and then queries are raised regarding the possible fertility sparing approaches. An older published article based on Surveillance, Epidemiology, and End Results (SEER) database showed that fertility sparing surgery such as ovarian and uterine-conserving surgery were safe with no actual effect on survival in young women who had stage IA and IC epithelial OC [18]. Concerning to lymph node dissection in fertility sparing surgery for early OC, Bogani et al. claimed that patients with bilateral ovarian involvement and serous OC are at high risk of having lymph node metastases despite the early pre- or intraoperative early stage disease. More specifically, among 290 patients who had lymph node dissection including pelvic and para-aortic lymphadenectomy, 14.5% patients were upstaged due to lymph node metastatic disease. Pelvic and para-aortic nodal metastases were observed in 7.6% and 14.5% patients. Lymph node involvement was observed in 18.9%, 2.7%, 13.8%, 17.4%, 7.3% and 20.8% patients with high-grade serous, low-grade-serous, endometrioid G1, endometrioid G2&3, clear cell and undifferentiated, histology, respectively [19]. However, systematic lymph node dissection has some major postoperative complications and its role in treatment of early stage OC patients who undergo fertility sparing surgery is controversial. Solution to this problem could be a randomized control trial; difficult to perform from an ethical aspect.

Regarding the role of *BRCA1* or *2* mutation presence and the adjustment of treatment, 8 out of 10 participants answered that molecular profiling of OC patients can differentiate their treatment. More specifically, PARP inhibitors (Olaparib) can be introduced as maintenance monotherapy in relapsed disease patients with *BRCA* mutation. Recently, SOLO-1 trial showed that among 391 patients with newly diagnosed advanced high-grade serous or endometrioid OC, primary peritoneal cancer, or fallopian-tube cancer (or a combination thereof) with a mutation in *BRCA1*, *BRCA2*, or both who underwent randomization, the use of maintenance therapy with olaparib provided a substantial benefit with regard to progression-free survival among women with newly diagnosed advanced OC and a *BRCA1/2* mutation, with a 70% lower risk of disease progression or death with olaparib than with placebo during a 41 month follow-up period [4, 20, 21].

The main purpose of this survey was the opinion of oncologists regarding the role of HIPEC in treatment of OC patients. Approximately 50% of the participants are positive in use of HIPEC in interval debulking surgery. A possible cornerstone in the management of OC patients is a recently published randomised trial which showed survival benefit. In a phase III trial that included 245 women who had at least stable disease after three cycles of neoadjuvant chemotherapy with carboplatin plus paclitaxel, the patients who underwent cytoreductive surgery with HIPEC experienced a significantly longer recurrence-free survival (hazard ratio [HR] 0.66; 95% CI, 0.50–0.87) and overall survival (OS) (HR: 0.67; 95% CI, 0.48–0.94) compared to those who underwent cytoreductive surgery alone. The rate of severe adverse events was similar in the two groups [6]. These results agree with a more recent protocol-based pilot study. This study revealed that IDS followed by paclitaxel-based HIPEC as a first-line treatment appears to be feasible and safe for the treatment of advanced-stage OC in a cohort of 65 patients [22]. A

HIPEC, is also used as a treatment option in patients suffered from OC recurrence. In our survey approximately 68% of the participants had a positive opinion about the utility of HIPEC in OC recurrence. Several retrospective studies, regarding the use of HIPEC in these patients, have been published. A prospective randomized phase III trial reported that patients who experienced OC recurrence and were treated with cytoreductive surgery followed by HIPEC and then systemic chemotherapy had mean survival 26.7 months in comparison with 13.4 months in patients who treated for ovarian recurrence with cytoreductive surgery and systemic chemotherapy only [8]. Also, in an article Iavazzo et al. reported their newer results regarding the use of HIPEC in patients with relapsed or residual OC. In this opinion letter, they shared that patients who treated with cytoreductive surgery, HIPEC and systemic chemotherapy had longer mean survival rate compared with patients underwent cytoreduction and systemic chemotherapy only [23]. Recently a meta-analysis of the published literature showed that OC patients with recurrence who received HIPEC exhibited significantly improved overall survival (HR=0.48, 95% CI=0.24–0.96, P<0.01) but not different disease-free survival (HR=0.59, 95% CI=0.33–1.08, P=0.09) [24]. Although, treatment of OC recurrence with cytoreductive surgery and HIPEC appears to be promising, the major aim of recurrent OC treatment is the complete cytoreduction.

HIPEC is also considered as a treatment option in patients undergoing primary cytoreductive surgery for OC. A randomized phase III clinical trial (ClinicalTrials.gov Identifier: NCT03842982) is now recruiting patients, in order to evaluate the efficacy of HIPEC in primary debulking surgery compared to interval debulking surgery. The primary results are expected in June of 2023 with anticipation [25].

As for any survey study, our study has several limitations that must be considered. As with all surveys the potential for inaccurate information because of response bias has to be acknowledged. All the participants shared their opinion by email. This makes the data collection more unreliable. Moreover some data are lacking, such as specific country contribution. Also, all the responders were medical oncologists, surgical oncologists or gynaecological oncologists, with an underrepresentation of general gynaecologists. Because of that distribution, there is the possibility of participants to be positively predisposed as for the use and the role of HIPEC in OC treatment.

Our survey adds to the published literature the current insight and opinion of Medical Oncologists, Surgical Oncologists and Gynaecologic Oncologists regarding the management and treatment of OC patients and the utility of HIPEC on those patients after the publication of some subversive trials such as SOLO-1 trial, LION trial and Van Driel’s et al. trial [6, 17, 21].

In conclusion, our survey confirmed some disparities in the current management of OC patients, including preoperative workup, optimal time of surgery, radicality of surgical procedure, possible role of lymphadenectomy, utility of HIPEC in primary surgery and in recurrent OC patients and adjuvant therapy regarding the *BRCA* status. This survey should stimulate new clinical trials, in order to investigate these controversial tactics in the management of OC patients.
